# Surface Sizing Treated MWCNTs and Its Effect on the Wettability, Interfacial Interaction and Flexural Properties of MWCNT/Epoxy Nanocomposites

**DOI:** 10.3390/nano8090680

**Published:** 2018-08-31

**Authors:** Qingjie Zhang, Xinfu Zhao, Gang Sui, Xiaoping Yang

**Affiliations:** 1 State Key Laboratory of Organic-Inorganic Composites, College of Materials Science and Engineering, Beijing University of Chemical Technology, Beijing 100029, China; qingjie05110@163.com (Q.Z.); zhaoxinfu1990@126.com (X.Z.); yangxp@mail.buct.edu.cn (X.Y.); 2 Department of Chemical Engineering, Tsinghua University, Beijing 100084, China

**Keywords:** nanocomposites, epoxy, MWCNTs, surface sizing, interfacial interaction

## Abstract

A surface-sizing technique was offered to take full advantage of multi-walled carbon nanotubes (MWCNTs) and epoxy resins. Two surface-sizing treated MWCNTs were obtained through a ball-milling treatment of amino-functionalized MWCNTs (MWCNT-NH_2_) with *n*-butyl glycidylether (BuGE) and benzyl glycidylether (BeGE). These were referred to as MWCNT-BuGE and MWCNT-BeGE. The results indicated that the surface sizing effectively enhanced wettability, dispersibility of MWCNTs in the epoxy resin. These ameliorating effects, along with improved interfacial interaction between MWCNT-BeGE containing benzene rings and the epoxy matrix, which can offer a more efficient local load-transfer from matrix to MWCNTs, as observed by a higher G-band shift in Raman spectrum under bending loads than that of MWCNT-BuGE reinforced ones. Correspondingly, MWCNT-BeGE/epoxy nanocomposites exhibited increasing flexural strength and modulus of 22.9% and 37.8% respectively compared with the neat epoxy, and 7.3% and 7.7% respectively compared with MWCNT-BuGE/epoxy nanocomposites with the same MWCNT content.

## 1. Introduction

Epoxy resin has been widely used as the polymer matrix of high performance composites in many engineering fields due to its three-dimensional cross-linked thermoset structures with outstanding mechanical and bonding properties [1,2,3]. Since the performance requirements of advanced composite materials have been continuously increased in some special industrial applications, such as aviation and aerospace industry, the mechanical properties of epoxy resin also need to be further improved. In addition to changing the chemical structure of the resin matrix, nano-enhancement is also an important technical mean to improve mechanical performances through the incorporation of nano-scale additives [4,5].

Among nano-fillers, carbon nanotubes (CNTs) exhibited high aspect ratios, low density and excellent strength and stiffness, making them excellent fillers for epoxy resins [6,7,8,9]. However, reinforcing effect of pristine CNTs on epoxy matrix was often limited by the strong van der Waals interaction and naturally high crystallinity, resulting in their agglomeration and weak interfacial interactions with the resin matrix [10]. Previous researchers often used hydroxy, carboxylic, amino, epoxy and silane functionalization, et al., to enhance the wettability, dispersibility, and surface reactivity of CNTs in a resin matrix [11,12,13,14,15,16,17].

Because amino groups can provide strong interactions with epoxy groups via nucleophilic ring-opening, by far, amino functionalization has been known as the most effective way of tackling interfacial interaction of CNTs in epoxy composites [18,19,20,21,22,23,24]. Khare et al. [22] proved that amido-amine functionalized CNTs facilitated the transfer of mechanical load across the matrix−filler interface, resulting in a ~50% improvement in the Young’s modulus compared with the neat cross-linked epoxy by the use of molecular dynamic (MD) simulation. Cha et al. [23] found that melamine functionalized CNT/epoxy nanocomposites exhibited enhancements of Young’s modulus by 64% and ultimate tensile strength by 22% compared with neat epoxy samples, due to covalent bonds with surface groups in the epoxy matrix via amino groups on melamine. Lavorgna et al. [24] found that silica nanoparticles and 3-amminopropryltriethoxy silane (APTES) functionalized CNTs acted synergistically to improve the storage modulus of nanocomposite of about 25% in the glassy region and 280% in the rubbery region compared with neat epoxy.

However, the solid CNTs still easily flocculated into bundles and agglomerated in the liquid resin matrix, due to a solid-liquid phase shift at the interface between CNTs and the matrix during the mixing process, resulting in a lower enhancement effect of the CNT-NH_2_ [23,25]. In addition, though traditional epoxy functionalized CNTs can overcome the problem of phase shift, their drawbacks are obvious, as follows: the functionalization method is complicated and non-ideal for large scale applications; some catalysts are introduced into the CNT preparation, which are difficult to remove in further applications [26].

The surface of commercial carbon fibers is often covered with a reactive sizing layer, which can prevent the fibers from damage through the manufacture process, and improve the interfacial wettability and adhesion between fibers and the resin matrix [27]. As one-dimensional materials, CNTs with large aspect ratios can also be treated using a surface sizing layer. However, so far, no studies have been reported in the literature for such a surface sizing technique and its influence on the wettability and dispersibility of CNTs in the epoxy resin, as well as interfacial interaction and mechanical reinforcement of the resulting CNT/epoxy nanocomposites.

In this present study, a surface-sizing processing was proposed to facilitate the preparation of multi-walled carbon nanotube (MWCNT) reinforced epoxy nanocomposites. Two surface sizing treated MWCNTs were obtained through a ball-milling treatment of amino functionalized MWCNT-NH_2_ with *n*-butyl glycidylether (BuGE) and benzyl glycidylether (BeGE). These were referred to as MWCNT-BuGE and MWCNT-BeGE. The effects of surface sizing on the wettability and dispersion of MWCNTs in epoxy matrix were determined and compared with control samples. Furthermore, Raman spectroscopy and MD simulation were used to study the interfacial interaction between MWCNTs and the resin matrix. Fractography and flexural properties of the resulting nanocomposites were also discussed with reference to this observation. The surface sizing technique mentioned in this study shows the potential industrial application prospect use to obtain high performance of MWCNT/epoxy nanocomposites.

## 2. Materials and Methods

**Figure 1:**
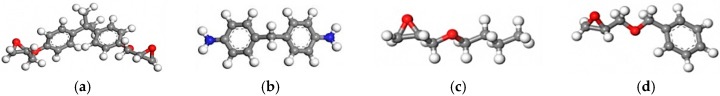
Simulated molecular structures employed in the molecular dynamic (MD) simulation: (**a**) diglycidyl ether of bisphenol A (DGEBA), (**b**) 4,4′-diaminodiphenyl methane (DDM), (**c**) *n*-butyl glycidylether (BuGE) and (**d**) benzyl glycidylether (BeGE), where carbon is shown in gray, oxygen in red, nitrogen in blue and hydrogen in white.

Diglycidyl ether of bisphenol A (DGEBA, epoxy value, 0.51) and the hardner 4,4′-diaminodiphenyl methane (DDM) were purchased from Tianjin Synthetic Material Research Institute (Tianjin, China). Epoxy diluents, *n*-butyl glycidylether (BuGE) and benzyl glycidylether (BeGE), were purchased from Aldrich chemical Co. (Milwaukee, WI, USA) as reactive mono-functional epoxy compounds. The simulated molecular structures of raw materials used in this study are shown in Figure 1. The MWCNT-NH_2_ (length, 1–5 μm; diameter, 10–20 nm) was synthesized through amidation of commercial carboxylated MWCNTs (purity, 95%; diameter, 10–20 nm, Chengdu Organic Chemical Co., Chengdu, China) with ethanediamine.

**Figure 2:**
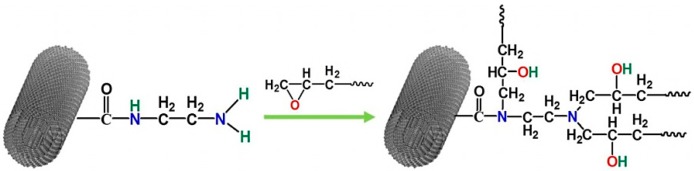
Reaction mechanism of surface sizing treated multi-walled carbon nanotubes (MWCNTs).

Two kinds of surface sizing treated MWCNTs were obtained by ball-milling (200 r/min, 4 h) MWCNT-NH_2_ and epoxy diluents BuGE and BeGE with a mass ratio of 1:2, and then let stand for 72 h at room temperature. After processing, the solid-liquid weight ratio of these surface sizing treated MWCNTs decreased to 1:1.5, which can be used as nano-fillers for the selected epoxy resin system. Besides, some surface sizing treated MWCNTs were rinsed for five times by using acetone to remove the adsorbed BuGE and BeGE, respectively, and then dried in an oven for later surface characterization. Because each amino group on the surface of MWCNT-NH_2_ had three reactive hydrogen atoms, ideally, there were three grafted molecule of diluents. The reaction mechanism of surface sizing treated MWCNTs is illustrated in Figure 2. MWCNT-NH_2_, MWCNT-BuGE and MWCNT-BeGE (2 mg) were dispersed in ethanol (10 mL) with ultrasonication for 30 min, respectively, and then MWCNT films were obtained by vacuum filtration of MWCNT dispersions via the cellulose filter with a pore size of 0.2 μm. The resulting MWCNT films were used in later wettability examinations. The 0.5 wt.% MWCNT-NH_2_, 1.25 wt.% MWCNT-BuGE and 1.25 wt.% MWCNT-BeGE (the contents of solid MWCNTs was 0.5 wt.%) were dispersed in DGEBA epoxy resin by mechanical stirring at room temperature for 10 h, respectively. After this, curing agents (mass ratio, DGEBA:DDM = 100:25) were added to the MWCNT/epoxy system, and the homogeneous mixture was degassed for 30 min at 80 °C under vacuum before curing. Then the resin systems were poured into a steel mold, and cured at 90 °C for 1 h, 130 °C for 2 h, and 180 °C for 2 h in an oven.

The MD simulation of interfacial interaction between the surface sizing treated MWCNTs and the resin matrix was performed using the Forcite module of Materials Studio (6.0, Accelrys Inc., San Diego, CA, USA). The condensed phase optimized molecular potential for atomistic simulation studies (COMPASS) force field was employed to analyze the intramolecular and intermolecular interactions with a constant number of molecules, constant volume, and constant temperature (NVT) ensemble at 300 K with no applied pressure [9]. A time step of 1 fs, an equilibration time of 0.5 ns, and a production run time of 30 ps were used in this simulation. The covalent interaction energy (*E_inter_*) between resin matrix and surface-sizing MWCNTs was calculated according to the Equation (1) [9]:

(1) $$E_{inter\;}=E_{total}-\left(E_{CNTs}+E_{resin\;matrix}\right)$$

where *E_total_* was the total potential energy between the surface-sizing MWCNTs and resin matrix pair via covalent interaction, and *E_CNTs_* was the potential energy of surface-sizing MWCNTs when the resin matrix was put at infinite distance, *E_resin matrix_* can be defined as the potential energy of resin matrix when the surface-sizing MWCNTs were put at infinite distance.

The MWCNT-NH_2_, MWCNT-BuGE and MWCNT-BeGE materials were analyzed and determined through high resolution transmission electron microscopy (HRTEM, Tecnai G2 F30 S, FEI Co., Hillsboro, OR, USA), transmission electron microscopy (TEM, JEM 3010, JEOL, Tokyo, Japan), Fourier-transform infrared spectroscopy (FT-IR, Nicolet 670, Nicolai Instruments, Madison, WI, USA), X-ray photoelectron spectroscopy (XPS, PHI-5300, Ulvac-Phi Inc., Chigasaki, Japan), thermogravimetric analysis (TGA, TA Q50, TA Instruments, New Castle, DE, USA) and Raman spectroscopy (TY-HR 800, HORIBA, Paris, France). In the thermogravimetric analysis, the heating rate was 10 °C/min from room temperature to 800 °C under N_2_ (FR = 40 mL/min). The Raman spectroscopy was obtained by using a near-infrared laser line (wavelength of 785 nm). Micro-mechanical behaviors of MWCNTs in resin matrix under bending loads were characterized by measuring a total of 676 points per image over a scan width and height of 5 μm × 5 μm. The contact angles of DGEBA on the MWCNT films were measured to investigate the changes in wettability on a goniometer (OCA20, DataPhysics, Stuttgart, Germany) at room temperature. The image analyzing software (Image J, 1.48, National Institutes of Health, Bethesda, MD, USA) was used to calculate the contact angle. Three key points were found first: left-end point, right-end point and vertex of the droplet. Then, the height (*h*) and width (2*r*) of droplet and the solid-liquid contact angle (*θ*) of the droplet can be obtained from the defined coordinates of these points. The dispersion performance of the MWCNTs in the resin matrix was observed using fractured, cured composite samples on a scanning electron microscope (SEM, JSM 7401F, JEOL, Tokyo, Japan) under 20 kV accelerating voltage. According to the standard test method ASTM D790, flexural tests of epoxy composites were performed on a material testing machine (WDS-10, Beijing Guance Jingdian Instrument Inc., Beijing, China) at the speed of 2 mm/min. Each data point was obtained from at least ten samples.

## 3. Results and Discussion

### 3.1. Surface Characterization of MWCNTs

**Figure 3:**
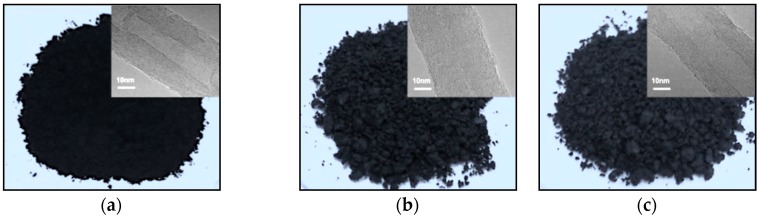

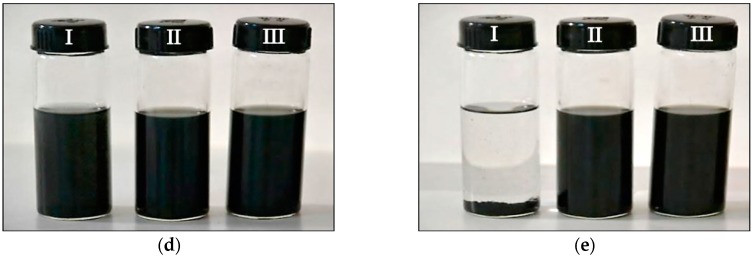
Optical images and high resolution transmission electron microscopy (HRTEM) (inset) micrographs of (**a**) MWCNT-NH_2_, (**b**) MWCNT-BuGE and (**c**) MWCNT-BeGE. The sedimentation behavior of (I) MWCNT-NH_2_, (II) MWCNT-BuGE and (III) MWCNT-BeGE in the ethanol solution after standing for (**d**) 1 h and (**e**) 24 h under 25 °C.

Figure 3a–c showed digital photographs and the corresponding HRTEM images (inset) of MWCNT-NH_2_, MWCNT-BuGE and MWCNT-BeGE. As shown, MWCNT-NH_2_ was solid powders, while MWCNT-BuGE and MWCNT-BeGE became semisolid particles after the surface sizing treatment. Compared with MWCNT-NH_2_, the structural integrity of MWCNT-BuGE and MWCNT-BeGE maintained well, indicating negligible damages to graphite sheet layer structure during surface sizing treatment (Figure 3a–c, inset). At the same time, the surfaces of both MWCNT-NH_2_ and surface sizing treated MWCNTs were covered by amorphous materials, which can be assigned to the chemical groups [28]. However, the difference between covering layers of these MWCNT samples did not distinguish well, due to the low molecular weight of chemical compounds.

Observing the dispersion stability of CNTs in an organic solvent has been considered a semi-qualitative method to judge success or failure of the surface modification of CNTs [29]. 1 mg/mL of MWCNT-NH_2_, MWCNT-BuGE and MWCNT-BeGE in absolute ethanol were ultrasonicated for 10 min, respectively, and their optical images were taken after standing for 1 h (Figure 3d) and 24 h (Figure 3e). MWCNT-NH_2_ was precipitated gradually, while MWCNT-BuGE and MWCNT-BeGE still retained dispersion stability after standing for 24 h, because the sizing processing changed the surface polarity of MWCNTs, preventing MWCNTs from re-aggregation.

**Figure 4:**
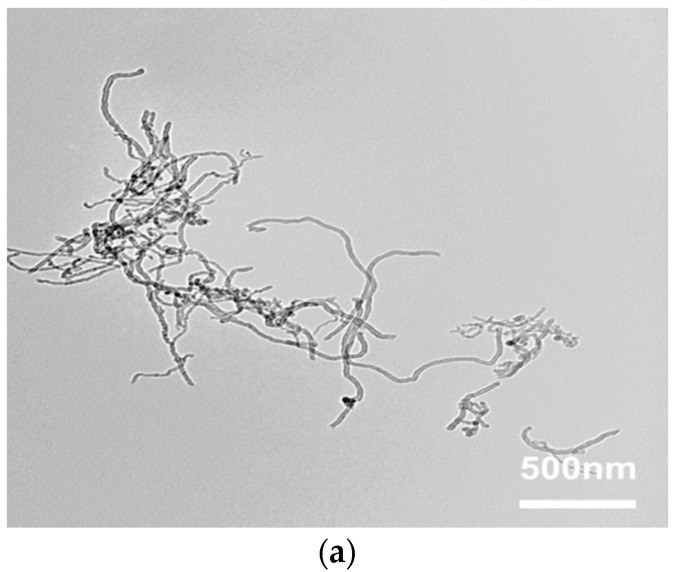

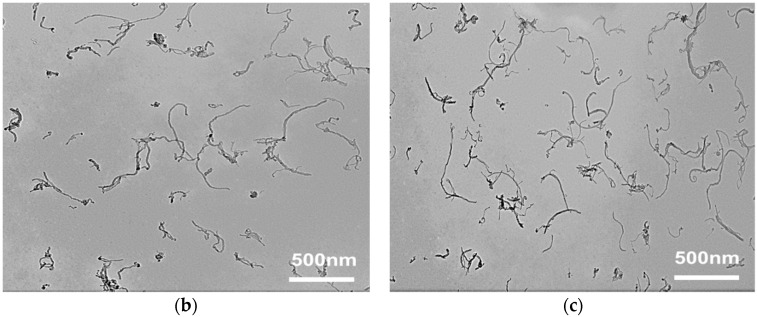
Transmission electron microscopy (TEM) photographs of (**a**) MWCNT-NH_2_, (**b**) MWCNT-BuGE and (**c**) MWCNT-BeGE.

Figure 4 shows TEM micrographs of MWCNT-NH_2_, MWCNT-BuGE and MWCNT-BeGE that further illustrate their dispersion states in ethanol as solvent. Small MWCNT agglomerates can be clearly seen in the sample of MWCNT-NH_2_ (Figure 4a). After the surface sizing process, the length of MWCNT-BuGE (Figure 4b) and MWCNT-BeGE (Figure 4c) was reduced from ~1–5 μm to ~0.5–1 μm, and no MWCNT agglomerates were observed. The surface sizing treatment led to the efficient debundling and dispersion of the MWCNTs in organic solvents.

**Figure 5:**
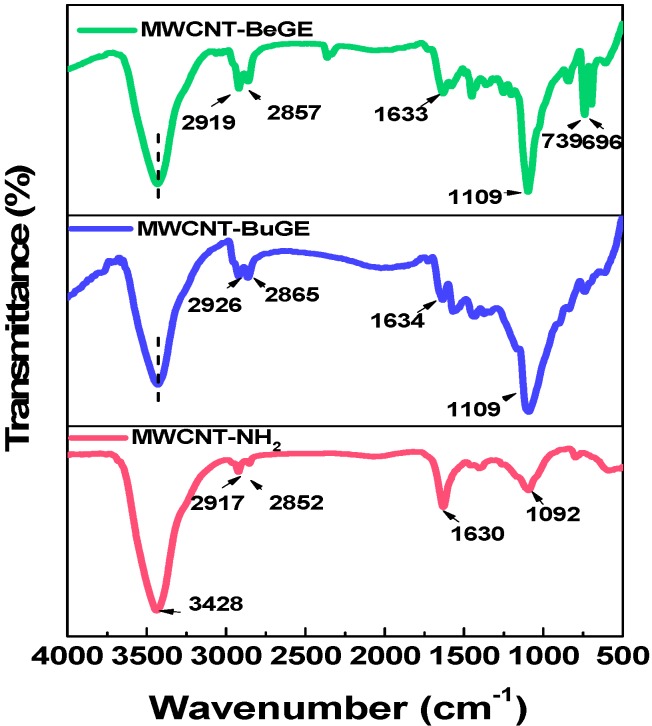
Fourier-transform infrared spectroscopy (FT-IR) spectra of MWCNT-NH_2_, MWCNT-BuGE and MWCNT-BeGE.

Figure 5 shows the FT-IR spectra of MWCNT-NH_2_, MWCNT-BuGE and MWCNT-BeGE. The 3428 cm^−1^ peak was originated from the O–H stretching vibration of water molecules which were present in the KBr as well as in carbon nanotubes [30]. The peaks of MWCNT-NH_2_ at 1630 cm^−1^ and 1092 cm^−1^ were ascribed to the stretching vibration of N–H, and C–N deriving from primary amino groups [25], respectively. Peaks at 2850–2926 cm^−1^ region were characteristics of symmetric and asymmetric stretching vibration of –CH_2_– groups, and their intensities were greatly enhanced in MWCNT-BuGE and MWCNT-BeGE materials. The introduction of BuGE and BeGE can be observed from the peak at 1109 cm^−1^, which was assigned to C–O–C stretching frequencies. Moreover, strong absorption peaks at 739 cm^−1^ and 696 cm^−1^ resulting from out of plane bending of the benzene ring were also observed in the MWCNT-BeGE.

**Figure 6:**
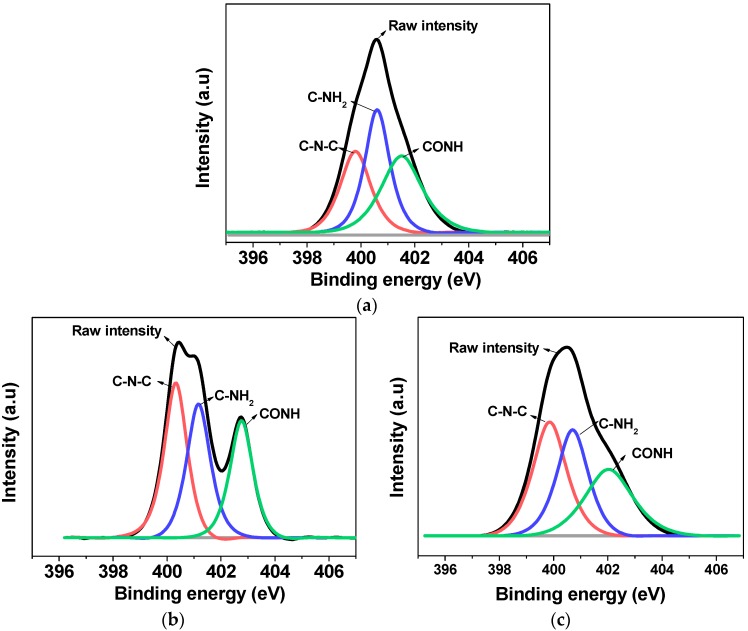
X-ray photoelectron spectroscopy (XPS) N1s curve fitting peak of (**a**) MWCNT-NH_2_, (**b**) MWCNT-BuGE and (**c**) MWCNT-BeGE.

To distinguish the adsorbed or chemically bonded surface molecules on the surface of MWCNTs, the identification of surface functionalities was performed through calculating the N1s peak area of MWCNT-NH_2_, MWCNT-BuGE and MWCNT-BeGE. Figure 6 shows XPS (N1s) curve fitting for three kinds of MWCNTs, and corresponding N1s peak area of each type of MWCNTs was shown in Table 1. N1s peak positions at 399.87 eV, 401.03 eV and 402.30 eV were attributed to the chemical bonds C–N–C, C–NH_2_ and –NH–C=O [25].

In the samples of the MWCNT-BuGE and MWCNT-BeGE, the peak area of C–N–C bond increased, while the corresponding peak area of C–NH_2_ bond decreased compared with the results for the MWCNT-NH_2_ (Table 1). These results suggested that new C–N–C chemical bonds were formed, which directly validated the chemical reaction between the epoxy diluent and the amino group of the MWCNT-NH_2_. The results of XPS analysis demonstrated that BuGE and BeGE have been successfully bonded to the surface of the MWCNT-NH_2_ through covalent bonds.

**Figure 7:**
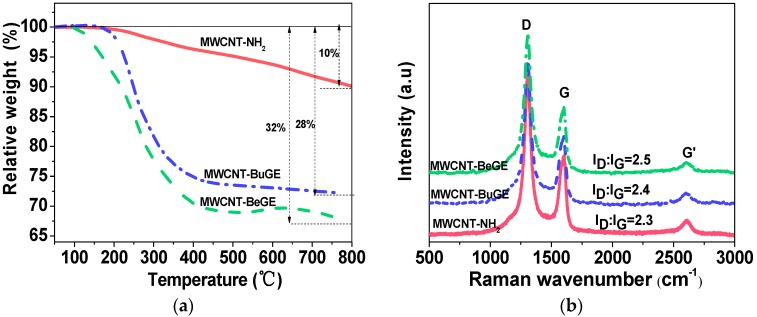
Thermogravimetric analysis (TGA) (**a**) and Raman spectra (**b**) of MWCNT-NH_2_, MWCNT-BuGE and MWCNT-BeGE.

Figure 7a shows the TGA analysis for MWCNT-NH_2_, MWCNT-BuGE and MWCNT-BeGE. The weight loss of MWCNT-NH_2_ was about 10% at 800 °C, due to the decomposition of the amino groups. The weight loss of the MWCNT-BuGE and MWCNT-BeGE was 32% and 28%, respectively. This increase in weight loss was attributed to the grafting of mono-functional epoxy molecules on the surface of MWCNT-NH_2_.

In addition, the weight loss of MWCNT-BuGE at 800 °C was higher than that of MWCNT-BeGE. This difference in weight loss between the MWCNT-BuGE and MWCNT-BeGE was attributed to the increased reactivity of the amino group (NH_2_–(CH_2_)_2_–NH–CO–) in the BuGE and BeGE molecules on the surface of MWCNTs. The ratio (*n*) of epoxy diluents to amino groups is roughly calculated using the Equation (2) [31]:

(2) $$n=\frac{\left(X_{e}-X_{a}\right)/M_{e}}{X_{a}/M_{a}}$$

where *X_e_* and *X_a_* is the weight loss of the MWCNT surface sizing and MWCNT-NH_2_; *M_e_* and *M_a_* is the molecular mass of BuGE (or BeGE) and the amino groups. The *n* of MWCNT-BuGE and MWCNT-BeGE was 1.5 and 1.0, indicating that BuGE reacted easier with the amine groups on the surface of MWCNT-NH_2_ than that of BeGE. Moreover, the remaining activated amine groups in the surface sizing still had the ability to participate into the crosslinking network via ring-opening reaction with epoxy groups.

Raman spectroscopy is one of the most important techniques for the analysis of the vibrational modes and the structure of carbon nanotubes [32,33]. Figure 7b shows Raman analysis results of the functionalized MWCNTs, where the peak positions at 1311 cm^−1^, 1601 cm^−1^ and 2608 cm^−1^ were attributed to the disorder-induced D band (in-plane vibrations of sp^3^ carbon), the G band (the crystalline graphitic and in-plane vibrations of sp^2^ carbon), and the G’ band (the second overtone of the D-band) [34]. The relative intensity ratio, *I*_D_/*I*_G_, was sensitive to physical and chemical processing of the CNTs, and it was an indication of the degree of amorphous carbon atoms to graphitic carbon atoms on the surface of the CNTs, which reflected the structural integrity of the graphite [33]. The *I*_D_/*I*_G_ of MWCNT-BuGE (2.4) and MWCNT-BeGE (2.5) was a little higher than that of the MWCNT-NH_2_ (2.3), suggesting that the surface sizing treatment had little negative effect on the structural integrity of the graphite, consistent with the observation of HRTEM analysis (Figure 3a–c).

### 3.2. Interfacial Analysis of MWCNT/Epoxy Nanocomposite

**Figure 8:**
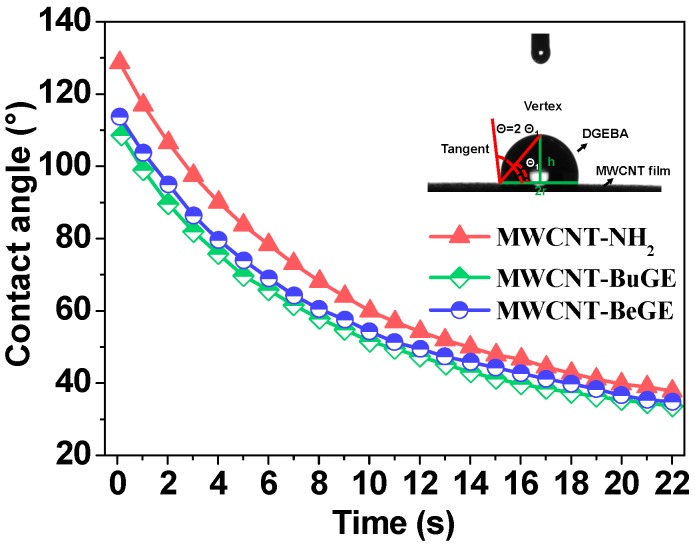
Dynamic contact angle of DGEBA on MWCNT-NH_2_, MWCNT-BuGE and MWCNT-BeGE films varies with time at room temperature (Inset image is the contact angle measurement process with side view of DGEBA on the MWCNT film).

For inorganic filler-reinforced polymeric composites, a good wettability is a primary prerequisite to rapidly construct interfaces between the fillers and the resin matrix [35,36]. Figure 8 shows contact angles changing with time of DGEBA on the MWCNT film at room temperature, which is used to evaluate the effect of surface sizing treatment on the MWCNT wettability with the resin matrix. As shown, the initial contact angle of DGEBA on the MWCNT-NH_2_ film was 129°. After the surface sizing treatment, the initial contact angles of DGEBA on the MWCNT-BuGE and MWCNT-BeGE films were down to around 110°, because the polarity of alkyl chain was smaller than that of the amino group. The liquid DGEBA spread over the MWCNT film due to the surface tension effect and the interfacial energy of the droplet, resulting in the decrease of contact angles with time. Dynamic contact angles of DGEBA on the MWCNT-BuGE and MWCNT-BeGE film were similar, but always smaller than that on the MWCNT-NH_2_ film. The results indicated that a better wettability with the epoxy resin can be obtained via surface-sizing treatment of MWCNT-NH_2_.

An adequate interfacial bonding strength can guarantee efficient load-transfer between the MWCNTs and resin matrix, and promote the comprehensive performance of the final nanocomposites [22,37]. When a strain occurred in a material system under the action of external force, the inter-atomic distances were changed, resulting in some changes of vibration frequency of some normal modes, which would be shown as a slight shift of Raman peak. The Raman G band of a better interfacial interaction between the MWCNTs and the epoxy matrix would shift to a higher wave number when the MWCNTs embedded in the epoxy resin were subjected to the strain [37]. Therefore, Raman G band shift can be used to assess the interfacial interaction between the MWCNTs and epoxy matrix.

**Figure 9:**
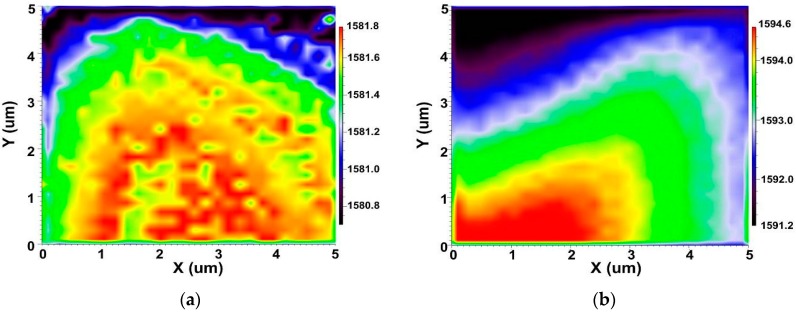

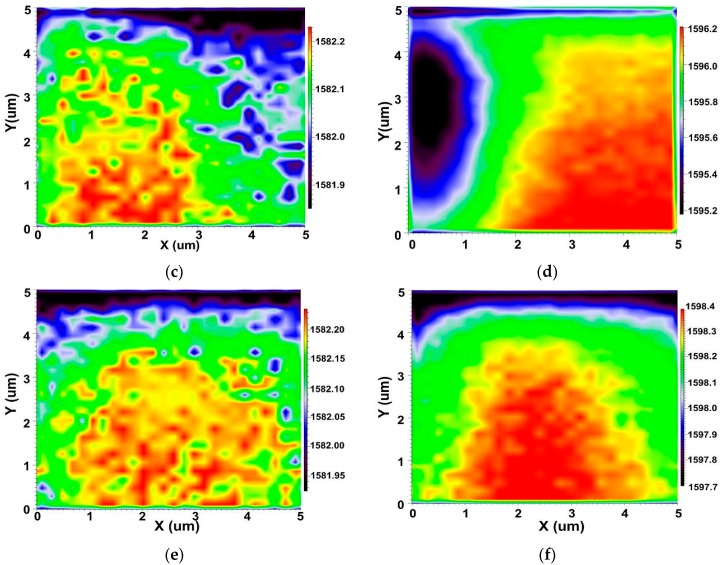
Raman mapping of G band of (**a**) MWCNT-NH_2_/epoxy nanocomposites, (**b**) MWCNT-NH_2_/epoxy nanocomposites with 1% bending load, (**c**) MWCNT-BuGE/epoxy nanocomposites, (**d**) MWCNT-BuGE/epoxy nanocomposites with 1% bending load, (**e**) MWCNT-BeGE/epoxy nanocomposites and (**f**) MWCNT-BeGE/epoxy nanocomposites with 1% bending load. Color-code: quantities related to MWCNTs are given in color, those of the epoxy matrix—in black.

Figure 9 shows the G band intensity distribution over the scanned area of cured DGEBA/DDM nanocomposites reinforced by MWCNT-NH_2_, MWCNT-BuGE and MWCNT-BeGE without or with 1% bending load at room temperature. There was no significant difference in the G band frequency (~1582.0) among the MWCNT-NH_2_, MWCNT-BuGE and MWCNT-BeGE before applying the external stress. However, when the 1% bending load was applied, the G band frequency of MWCNT-BuGE (1596.2) and MWCNT-BeGE (1598.4) shifted to a higher wave number than that of MWCNT-NH_2_ (1594.6), indicating the effective stress transfer from the resin matrix to the surface sizing treated MWCNTs at their interfaces. The G band frequency of MWCNT-BeGE/epoxy nanocomposites was higher than that of MWCNT-BuGE/epoxy nanocomposites owing to the stronger interfacial interaction among the MWCNT-BeGE and the epoxy matrix.

**Figure 10:**
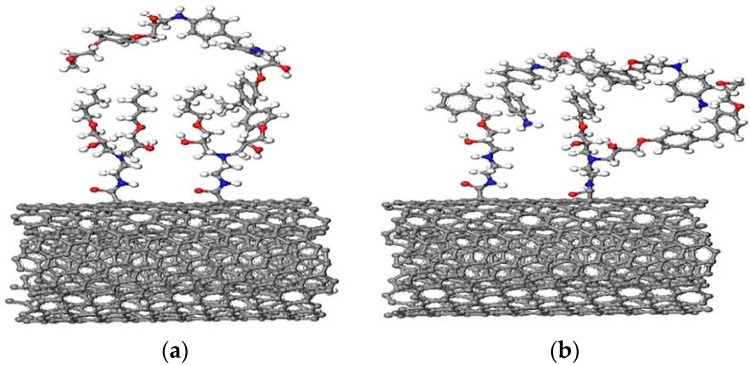

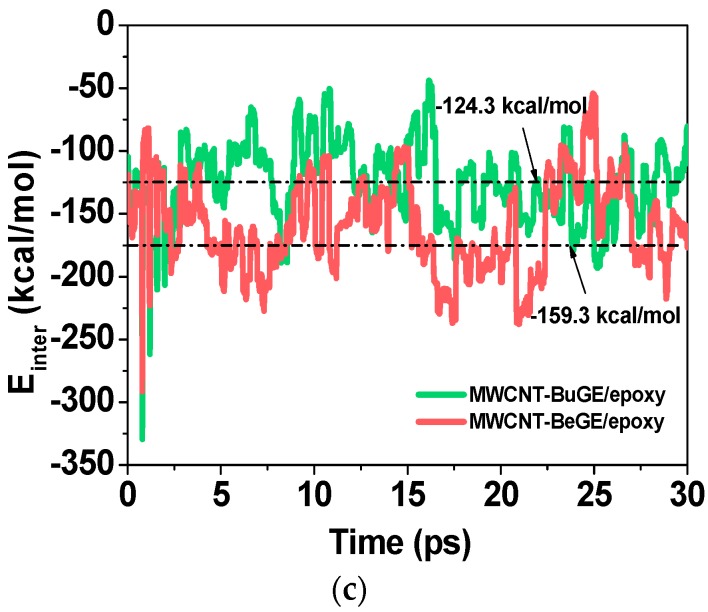
Schematics of interfacial microstructure of (**a**) MWCNT-BuGE/epoxy nanocomposites and (**b**) MWCNT-BeGE/epoxy nanocomposites; (**c**) the calculated intermolecular interaction energy of MWCNT-BuGE/epoxy and MWCNT-BeGE/epoxy during the relaxation process.

Figure 10 shows relaxed conformations of the modeled MWCNT-BuGE/DGEBA/DDM (Figure 10a) and MWCNT-BeGE/DGEBA/DDM (Figure 10b) at 300 K using the MD simulation, and the evolution of interaction energy between each model chain and surface sizing treated MWCNTs during the relaxation process (Figure 10c). MWCNT-BeGE/DGEBA/DDM exhibited a higher average interaction energy (−124.6 kcal/mol) than that of the MWCNT-BuGE/DGEBA/DDM (−159.3 kcal/mol), which was in accordance with the results of the Raman spectral analysis above. The improved interfacial interaction can be contributed to the formation π-π stacking between the aromatic rings in the MWCNT-BeGE backbone and the resin matrix [9,38].

### 3.3. Fracture Morphology and Mechanical Analysis of MWCNT/Epoxy Nanocomposite

**Figure 11:**
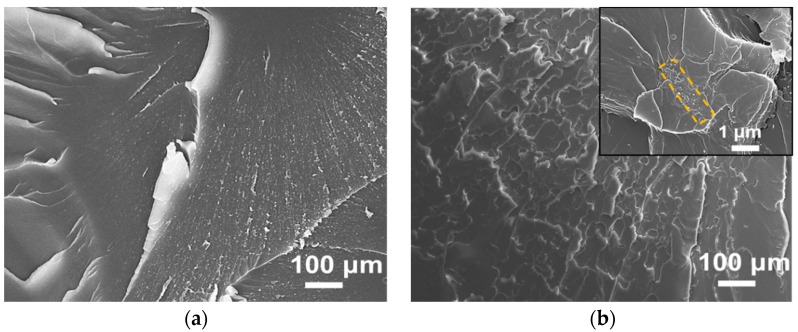

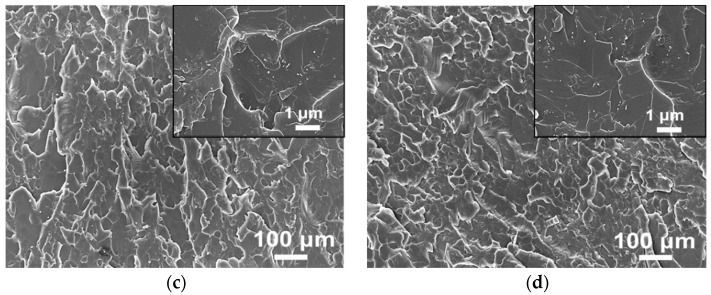
Scanning electron microscope (SEM) micrographs of fracture surface: (**a**) neat epoxy, (**b**) MWCNT-NH_2_/epoxy, (**c**) MWCNT-BuGE/epoxy and (**d**) MWCNT-BeGE/epoxy. Inset, high-magnification micrographs of dispersed MWCNTs in epoxy nanocomposites.

The fracture surfaces of neat resin and nanocomposites containing MWCNT-NH_2_, MWCNT-BuGE and MWCNT-BeGE were compared using SEM, as shown in Figure 11. Small MWCNT agglomerates were clearly seen from the sample containing MWCNT-NH_2_ (Figure 11b, inset), while these agglomerates were almost absent in the sample containing MWCNT-BuGE (Figure 11c, inset) and MWCNT-BeGE (Figure 11d, inset). This result indicated that the surface sizing treated MWCNTs further improved the dispersion effect of MWCNT-NH_2_ in the resin matrix. The surface sizing weakened the van der Waals’ (vdW) force between the MWCNTs and enhanced the chemical compatibility with epoxy matrix, and thus led to the efficient debundling and satisfactory dispersion of MWCNTs in the nanocomposites.

The neat epoxy exhibited a smooth fracture surface, revealing the typical brittle fracture with poor resistance towards cracking or rupturing. By contrast, the fracture surfaces of the nanocomposites exhibited ridges, rivers and plastic deformation features. This was primarily because the presence of MWCNTs prevented cracks from propagating, increased the area of the fracture surface and provided a high resistance to fracture [39]. Compared with the MWCNT-NH_2_, surface sizing treated MWCNTs showed a more evident reinforcement role in the resulting epoxy nanocomposites. Especially, the MWCNT-BeGE reinforced nanocomposites showed rather intricate and tortuous crack propagation, owing to the improved MWCNT dispersion, and interfacial energy between the MWCNT-BeGE and resin matrix promoted the load transfer effectively.

**Figure 12:**
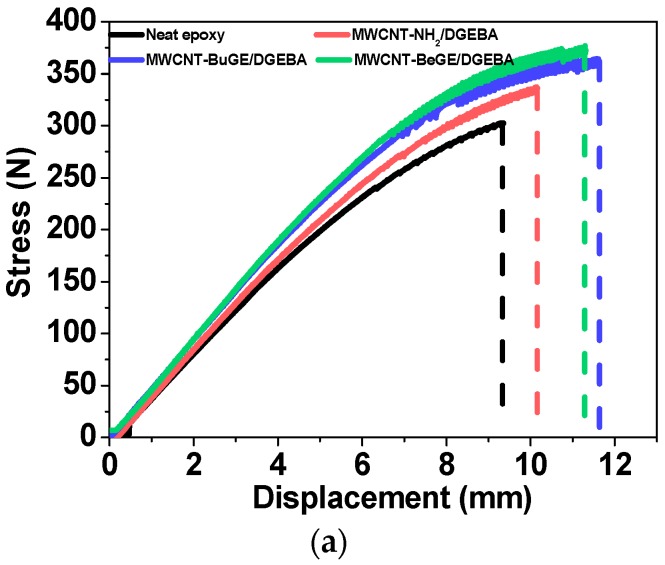

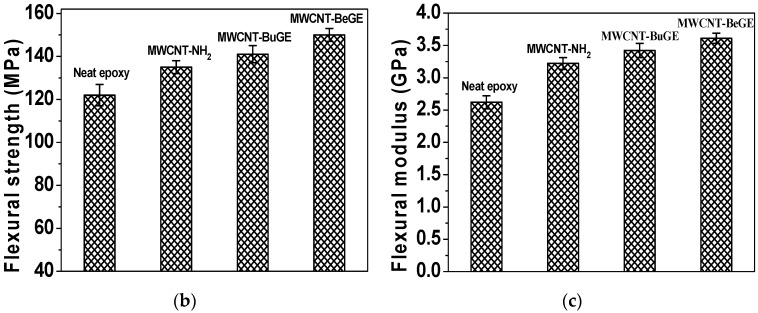
(**a**) Typical flexural stress-displacement curves, (**b**) the corresponding flexural strength and (**c**) flexural modulus of different nanocomposites.

Flexural properties of MWCNT/epoxy resin composites were closely related to the MWCNT dispersion and interfacial interactions between the MWCNTs and resin matrix [25,39,40,41]. Figure 12a shows typical flexural stress-displacement curves of DGEBA/DDM reinforced by MWCNT-NH_2_, MWCNT-BuGE and MWCNT-BeGE, and the average flexural strength and modulus were summarized in Figure 12b,c. Flexural strength and modulus of the neat resin were found to be 122 MPa and 2.6 GPa, while these properties of MWCNT-NH_2_ reinforced nanocomposites reached 135 MPa and 3.2 GPa, with an increase of 11.0% and 23.1%. By contrast, the surface sizing treated MWCNT/epoxy nanocomposites showed better flexural properties owing to the improved dispersion and wettability performance of epoxy-sizing MWCNTs compared with the MWCNT-NH_2_. The flexural strength and modulus of MWCNT-BeGE/epoxy nanocomposites can reach 150 MPa and 3.6 GPa, with an increase of 22.9% and 37.8% compared with the neat epoxy, and 7.3% and 7.7% compared with MWCNT-BuGE/epoxy nanocomposites with the same MWCNT content, indicating that the surface sizing containing benzene rings offered a more efficient local load-transfer between MWCNTs and the epoxy matrix.

The comparison of flexural properties and their increases between this study and other researches for MWCNT/DGEBA systems is summarized in Table 2. Compared with control samples, increasing flexural strength from 12.1% to 26.7% and modulus from 17.6% to 21.7% of MWCNT/epoxy nanocomposites was observed in previous articles, which were inferior to synthetically reinforcing effect of surface sizing treated MWCNTs.

From the analysis above, the surface sizing treatment can effectively improve the wettability and dispersion of MWCNTs in the epoxy matrix. Moreover, a higher interfacial energy between the MWCNTs and epoxy matrix can improve the local load-transfer from weak resin matrix to the strong MWCNTs through the structural design of surface sizing. The surface sizing treatment is an effective process method to take full advantage of MWCNTs and epoxy resins, and has shown a good application prospect.

## 4. Conclusions

In this study, through a ball-milling treatment with BuGE and BeGE, the MWCNT-NH_2_ material was converted into two surface sizing treated MWCNTs: MWCNT-BuGE and MWCNT-BeGE. The surface sizing treatment effectively enhanced wettability, dispersibility and mechanical reinforcement of MWCNTs in the epoxy resin compared with MWCNT-NH_2_. The results of MD simulation and Raman spectroscopy indicated that the surface sizing containing benzene rings (MWCNT-BeGE) improved the interaction energy, and offered a more efficient local load-transfer between MWCNTs and the epoxy matrix, as observed by a higher G-band shift in Raman spectrum under bending loads than that of MWCNT-BuGE reinforced ones. As a consequence, the flexural strength and modulus of MWCNT-BeGE/epoxy nanocomposites reached 150 MPa and 3.6 GPa, with an increase of 22.9% and 37.8% compared with the neat epoxy, and 7.3% and 7.7% compared with MWCNT-BuGE/epoxy nanocomposites with the same MWCNT content.

## Figures and Tables

**Table 1:** N1s peak area of MWCNT-NH_2_, MWCNT-BuGE and MWCNT-BeGE.

Samples	C–N–C (%)	C–NH_2_ (%)	–NH–C=O (%)
MWCNT-NH_2_	31.4	35.0	33.6
MWCNT-BuGE	38.0	33.5	28.5
MWCNT-BeGE	36.7	31.9	31.4

**Table 2:** Comparison of flexural properties between this study and other researches for MWCNT/DGEBA systems.

CNT Type	Content (wt.%)	Flexural Strength/Mpa (Increase, %)		Flexural Modulus/GPa (Increase, %)		Reference
		Control Sample	Composites	Control Sample	Composites	
MWCNT-NH_2_	0.5	107	128 (19.6%)	2.92	3.51 (20.2%)	[25]
MWCNT-NH_2_	0.5	90	102 (13.3%)	1.88	2.21 (17.6%)	[39]
MWCNT-NH_2_	0.6	86	109 (26.7%)	2.21	2.69 (21.7%)	[40]
MWCNT derivatives	1	109	122 (12.1%)	-	-	[41]
MWCNT-NH_2_	0.5	122	135 (10.7%)	2.62	3.22 (22.9%)	This study
MWCNT-BuGE	0.5	122	141 (15.6%)	2.62	3.41 (30.1%)	This study
MWCNT-BeGE	0.5	122	150 (22.9%)	2.62	3.61 (37.8%)	This study
